# Applying multi-theory model to determine intentions to breast self-awareness among female health workers in Kabul, Afghanistan

**DOI:** 10.3389/fpubh.2026.1784908

**Published:** 2026-04-22

**Authors:** Masouma Mohammadi, Farkhondeh Amin Shokravi, Hasan Shahbazi

**Affiliations:** Department of Health Education and Health Promotion, Faculty of Medical Sciences, Tarbiat Modares University, Tehran, Iran

**Keywords:** breast self-awareness, health workers, initiation, multi-theory model, sustenance

## Abstract

**Background:**

In women, breast cancer has the highest incidence compared to other cancers. Breast self-awareness may facilitate early detection of cancer through self-examination. This study aimed to apply the Multi-Theory Model (MTM) of Health Behavior Change to determine breast self-awareness intentions among female health workers in the western region of Kabul.

**Method:**

Female health workers from Western Kabul city were invited to participate in this cross-sectional study by visiting different health centers using a convenience sampling method. A 37-item researcher-developed questionnaire with established validity and reliability based on MTM was used. In this study, 130 eligible participants completed the questionnaire. Multiple linear regression was used to explain breast self-awareness behavior.

**Results:**

The mean age of the participants was 26.46 years (SD = 5.2). The study found that changes in the physical environment (β = 0.095, *p* = 0.012) and behavioral confidence (β = 0.053, *p* = 0.010) were significantly associated with initiation of breast self-awareness. In addition, intention to maintain breast self-awareness behavior was significantly associated with practice for change (beta = 0.161, *p* < 0.001) and changes in the social environment (beta = 0.077, *p* = 0.008).

**Conclusion:**

The findings suggest that constructs of the Multi-Theory Model are associated with initiation and maintenance of breast self-awareness behavior. These results provide preliminary evidence supporting the relevance of the MTM framework in this population. Future interventions may benefit from incorporating these constructs to promote breast self-awareness for breast cancer prevention.

## Introduction

Cancer is the second leading cause of death among non-communicable diseases (1). In women, breast cancer is defined as a malignant tumor arising from breast cells (2). Breast cancer is the most common cancer among women in both developed and developing countries. Globally, More than 60% of cancer cases occur in Africa, Asia, Central and South America, and approximately 70% of cancer-related deaths occur in these regions, reflecting substantial disparities in prevention, early detection, and access to care (3). Almost half of all cancer cases occur in Asia, a quarter in Europe and the remainder in African and American countries. Notably, nearly 50% of breast cancer cases and deaths occur in less developed countries, emphasizing the need for effective, context-specific preventive strategies (3).

The incidence rate of breast cancer in the United States increased by 5% per year from 2010 to 2019 (4). Pakistan has a high incidence and mortality rate for breast cancer, with 14% of all cancers in the country attributable to breast cancer (5). Breast cancer is the most common cancer among Iranian women and the fifth leading cause of cancer-related deaths (6). In Afghanistan, cancer cases are projected to increase by 54% between 2018 and 2030, posing a significant public health challenge (7). Breast cancer is the most commonly reported cancer in Afghanistan and occurs at a younger age than in other countries (8, 9). Afghan women have poor breast cancer screening practices, highlighting the need for increased awareness and access to screening programs (10). Breast cancer is the most common breast lesion diagnosed in Afghan women, accounting for 24% of cases (8).

Another study found that breast cancer accounts for 45.8% of cancers diagnosed in Afghan women (11). In recent years have increasingly emphasized breast self-awareness rather than structured breast self-examination, as breast self-awareness focuses on a woman's familiarity with the normal appearance and feel of her breasts rather than adherence to a specific technique or schedule. Breast self-awareness is closely aligned with health education and health promotion approaches, as it relies on knowledge acquisition, empowerment, and sustained behavioral engagement, particularly in settings where access to organized screening programs is limited. Primary health care services, effective educational programs, and health professionals play a crucial role in the detection and prevention of cancer (12–18). In developing countries, early diagnosis and timely treatment—often supported by education-based interventions—are directly associated with improved survival among breast cancer patients (19–21). Understanding the factors that influence the adoption and continuation of such preventive behaviors requires the use of theory-based frameworks that allow for behavioral prediction and intervention planning. As a health behavior theory, the Multi-Theory Model (MTM) of health behavior change uses a fourth-generation framework to initiate and maintain behavior change. This model has been tested and validated in influencing a wide range of health behaviors in a variety of settings around the world (22–32). Given its emphasis on empowerment, environmental facilitation, and sustained behavior change, MTM is particularly relevant for theory-based health education and health promotion interventions.

**Figure 1:**
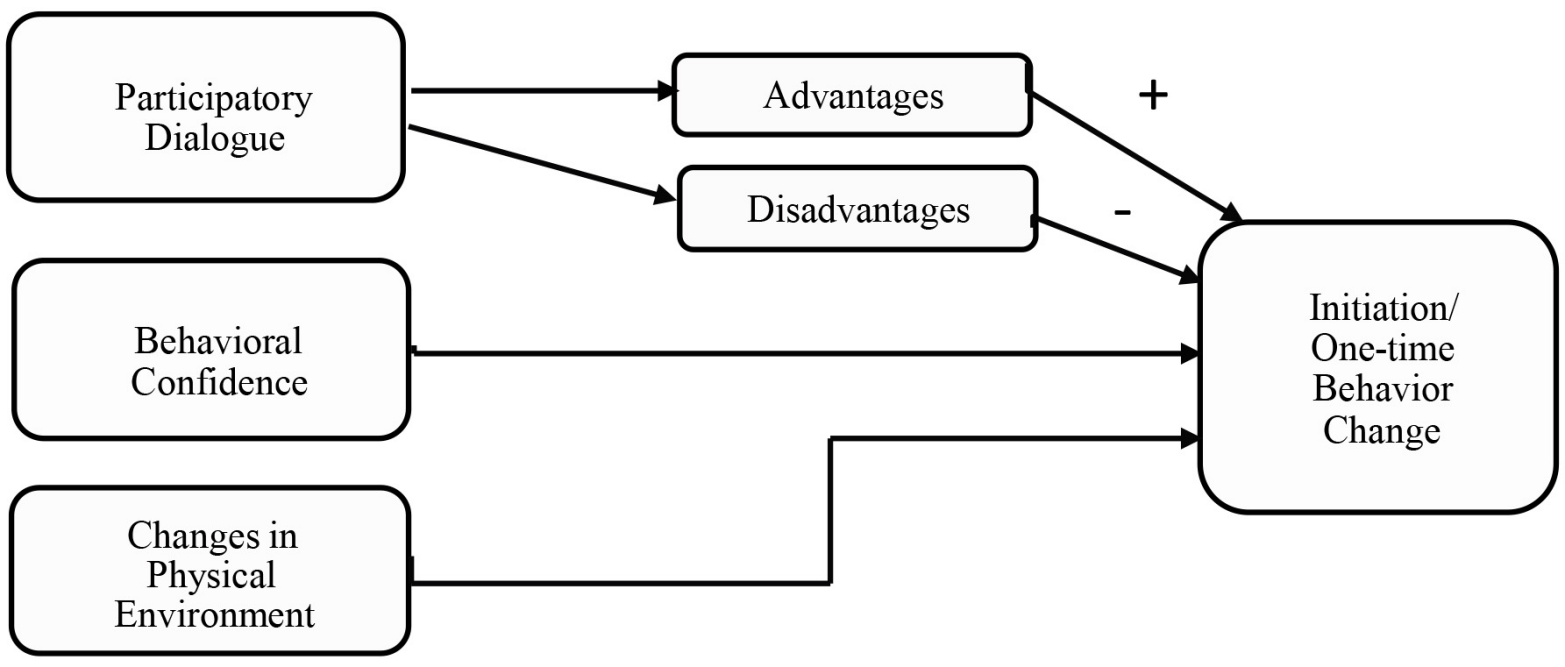
Constructs of breast self-awareness initiation in the multi-theory model (MTM).

As shown in Figure 1, the initiation phase of the Multi-Theory Model (MTM) of behavior change consists of three constructs:

Participatory dialogue—weighing up the pros and cons of health behavior change.

Behavioral confidence—the individual's belief in their ability to make the behavior change.

Changes in the physical environment—the availability of the necessary resources to facilitate behavior (33, 34).

**Figure 2:**
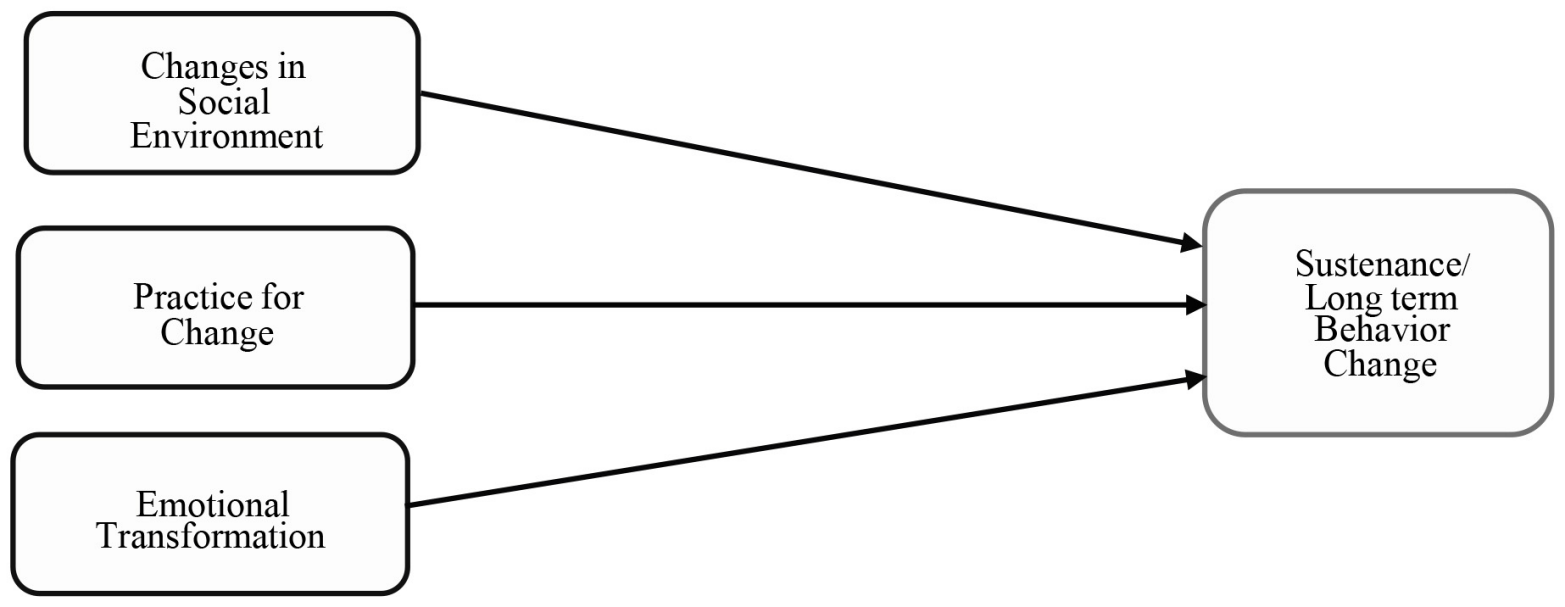
Constructs of breast self-awareness sustenance in MTM.

The constructs for the maintenance phase of behavior change can be categorized into three types (see Figure 2):

Emotional transformation—transforming emotions into goals that drive behavior change.

Practice for Change—developing a habit of change and integrating it into a lifestyle.

Changes in the social environment—gaining social support to sustain health behavior change (33, 34).

As there have been no studies on breast self-awareness using the Multi-Theory Model (MTM) of health behavior change in Afghanistan, we aimed to use this model to assess intention to initiate and maintain breast self-awareness behavior.

## Methods

This cross-sectional study was conducted between April and July 2021. Participants were included based on the following eligibility criteria: employed at a health center, over 20 years of age, no history of breast disease, not pregnant, and completed an informed consent form to participate in the study. Allowing for a drop-out rate of 7%, the total sample size was estimated to be 130 participants, assuming an alpha of 0.05, a power of 0.90, an effect size of 0.1, and a response rate of 80%. Each model included three predictors and five auxiliary variables, calculated using G^*^Power 3.1 software (Heinrich Heine University, Düsseldorf, Germany) (35). The minimum required sample size was 108 participants, and 130 participants were finally included, ensuring sufficient power for the planned analyses despite minor potential dropouts.

The independent variables in the initiation phase of behavior change include participatory dialogue, behavioral confidence and changes in the physical environment. In the maintenance phase, the independent variables are emotional change, practice for change and changes in the social environment. The dependent variables in the Multi-Theory Model (MTM) are intention to initiate and maintenance of health behavior change (breast self-awareness).

This study used a researcher-developed self-report questionnaire consisting of 37 questions (eight demographic questions and 29 model-related questions). The first eight questions were related to breast health practices (e.g., history of breast care and self-awareness) and demographic factors (e.g., age, education, marital status, employment status and income).

The remaining 29 questions were designed to assess constructs related to two models:

a) Initiation model, which included participatory dialogue, behavioral confidence and changes in the physical environment.
b) Maintenance model, which included emotional transformation, practice for change, and changes in the social environment (36).

The questionnaire was administered in a paper-based format within the healthcare facilities where participants were employed. All participants were health workers, and therefore, the questionnaire was fully self-administered. Data collection was conducted in a designated private room to ensure confidentiality and minimize external influence. No supervisors, administrative authorities, or senior staff members were present during completion of the questionnaire. Participants were informed that their responses were anonymous, no identifying information was collected, and participation was entirely voluntary.

### Validity and reliability

The validity and reliability of the questionnaire were assessed. To assess face validity, the questionnaire was tested on 30 health workers to determine whether the items were clear and understandable. Minor revisions were made based on their feedback to improve clarity and comprehension. Subsequently, the questionnaire was sent to Professor Manoj Sharma, the creator of the Multi-Theory Model (MTM), for expert review. His feedback confirmed that the questionnaire appropriately reflected MTM constructs, ensuring content relevance and theoretical alignment.

For reliability, internal consistency was measured for the variables related to the constructs of the initiation and maintenance phases of health behavior change in the Multi-Theory Model (MTM). Cranach's alpha values were found to be 0.86 and 0.89, respectively.

It is important to note that initiation of breast self-awareness behavior involves the decision to become aware of breast health, whereas maintenance of the behavior refers to the consistent practice of self-awareness (36). For both initiation and maintenance behaviors, responses were rated on a scale from “not at all likely” (0) to “somewhat likely” (1), “moderately likely” (2), “very likely” (3) and “completely likely” (4) (37). The possible scores ranged from 0 to 20.

### The multi-theory model (MTM) of health behavior change

1) Initiation of breast self-awareness behavior

a. Participatory dialogue provides information about breast self-awareness and its advantages and disadvantages. The benefits of breast self-awareness were assessed through five questions covering aspects such as good breast health, feel empowered in health care, take the time to take self-care of your breast health, being relaxed, and be notified sooner if there is a disease. Similarly, the disadvantages were discussed using five questions covering concerns such as having more worries, being afraid of developing the disease, not having time to do it, not overcoming your fears, and having unnecessary medical consultations (37). The possible scores ranged from 0 to 20.

It was hypothesized that higher scores for advantages and lower scores for disadvantages would be associated with initiation of breast self-awareness. By subtracting the disadvantages score from the advantages score, the participatory dialogue scores ranged from −20 to +20 (37).

b) Behavioral confidence

Behavioral confidence was assessed using a five-item assessment designed to determine an individual's confidence in performing breast self-awareness. The items included:

Confidence in performing breast self-awareness this week.

Confidence in performing breast self-awareness this week, despite being busy.

Confidence in performing breast self-awareness this week with ease.

Confidence in performing breast self-awareness this week without anxiety.

Confidence in performing breast self-awareness this week thoroughly without missing anything.

Answers were rated on a five-point scale:

Not at all confident (0), Somewhat confident (1), Moderately confident (2), Very confident (3), Completely confident (4).

The possible score ranged from 0 to 20. Higher scores indicated a greater likelihood of initiating breast self-awareness (37).

c) Changes in the physical environment

Changes in the physical environment were assessed using three questions focusing on the environment:

The ability to consider a location for breast self-awareness this week.

The ability to approach breast health care facilities this week if in doubt.

The ability to afford breast health care if needed.

Responses to each question were rated on a five-point scale:

Not at all confident (0) somewhat confident (1) Moderately confident (2) Very confident (3) Completely confident (4).

Possible scores ranged from 0 to 12. Higher scores indicated a greater likelihood of initiating a breast self-awareness (37).

2) Maintenance of breast self-awareness behavior

a) Emotional transformation

Emotional transformation was assessed by three questions focusing on emotions that facilitate breast self-awareness:

The ability to direct your emotions/feelings to the goal of performing breast self-awareness every week.

The ability to motivate yourself to take care of your breast health every week.

The ability to overcome your self-doubts/fears in accomplishing the goal of breast health care every week.

Each question was rated on a five-point scale as follows:

Not at all confident (0), Somewhat confident (1), Moderately confident (2), Very confident (3), Totally confident (4).

The total score ranged from 0 to 12. Higher scores indicated a greater likelihood of continuing breast self-awareness (37).

b) Practice for change

Practice for change was assessed using three items related to breast self-awareness maintenance:

The ability to use a self-diary or reminder system to monitor the time of your breast self-awareness every week.

The ability to continue to take care of your breast health every week if you encounter barriers.

The ability to change your plans for breast health care if you face difficulties.

Responses for each item were rated on a five-point scale:

Not at all confident (0), Somewhat confident (1), Moderately confident (2), Very confident (3), Totally confident (4).

The total score ranged from 0 to 12. Higher scores indicated a greater likelihood of consistently maintaining breast self-awareness (37).

C) Social environment changes were assessed using three key indicators to determine the likelihood of receiving support from family members, friends, and healthcare providers:

1. How sure are you that you can get the help of a family member to remind you for breast self-awareness every week?
2. How sure are you that you can get the help of a friend to provide you with support for breast self-awareness every week?
3. How sure are you that you can get the help of a health professional if you have doubts about your breast self-awareness every week

Responses were rated on a scale from 0 (“Not at all confident”) to 4 (“Completely confident”), with total possible scores ranging from 0 to 12. Higher scores indicated a greater likelihood of successfully maintaining breast self-awareness (36).

### Ethical considerations

This study received ethical approval from the Research Ethics Committee of Tarbiat Modares University (IR.MODARES.REC.1399.257). Participants were fully informed about the objectives of the study at the outset. No personal information was collected, and participation was entirely voluntary. Written informed consent was obtained from all participants prior to the commencement of the study.

### Statistical analysis

The data collected were analyzed using IBM SPSS version 26 (IBM Corp., Armonk, NY, USA). Continuous variables were presented as mean ± standard deviation. Stepwise regression analysis was performed to identify the optimal predictors of change in breast self-awareness behavior, particularly in terms of initiation and maintenance of the behavior, controlling for demographic characteristics. Multicollinearity was assessed using collinearity statistics, with tolerance values ranging from 0.651 to 0.877, indicating no significant multicollinearity among predictors. The regression models were statistically significant, and relationships between variables were consistent with theoretical expectations. Although detailed residual diagnostics (e.g., normality, homoscedasticity) were not formally assessed, multicollinearity diagnostics indicated no issues, and the overall data structure and correlation patterns do not suggest major violations of regression assumptions. A significance level of *p* < 0.05 was used.

## Result

Participants who met the inclusion criteria completed the paper-based questionnaire. The mean age of the participants was 26.46 ± 5.1 years. Their mean weekly working hours were 52.7 ± 23.16 h. The majority of participants had an income between 10,000 and 20,000 Afghanis, 48.5% had a bachelor's degree and 65.4% were married. Detailed demographic information on the participants is presented in Table 1.

**Table 1:** Demographic characteristics of study participants (*n* = 130).

Variable		Mean ±SD or *N* (%)
Age		26.46 ± 5.1
Weekly working hours		52.7 ± 23.16
Income (in 1,000 Afghanis)	Less than 10	39 (30%)
	10–20	47 (36.2%)
	20–50	34 (26.2%)
	50 to 100	15 (6.2%)
	More than 100	4 (1.5%)
Education	Upper diploma	58 (44.6%)
	Bachelor	63 (48.5%)
	Postgraduate	9 (6.9%)
Marital status	Married	85 (65.4%)
	Single	45 (34.6%)

Data are presented as mean ± SD or frequency (percentage).

Table 2 provides details of all constructs in the multi-theory model, including descriptive statistics and reliability calculations. Using a five-point Likert scale, the mean and standard deviation for the intention to initiate behavior was 2.7 ± 1.20. In the initiation model, the mean and standard deviation were 4.01 ± 6.21 for participatory dialogue, 14.3 ± 5.68 for behavioral confidence, and 8.3 ± 3.2 for changes in the physical environment.

**Table 2:** Descriptive statistics and reliability of MTM constructs (*n* = 130).

Constructs	Possible range	Observed range	Mean ±SD	Cronbach's alpha
Initiation of behavior	0–4	0–4	2.7 ± 1.20	0.864
Participatory dialogue	−20 to 20	−10 to 14	4.01 ± 6.21	—
Behavioral confidence	0–20	0–20	14.3 ± 5.68	—
Physical environment changes	0–12	0–12	8.3 ± 3.2	—
Maintenance of behavior	0–4	0–4	3.1 ± 1.1	0.892
Emotional transformation	0–12	0–12	8.9 ± 3.3	—
Practice for change	0–12	0–12	8.8 ± 3.2	—
Social environment changes	0–12	0–12	8.6 ± 3.3	—

Values represent possible and observed ranges, means, standard deviations, and Cronbach's alpha.

A multiple linear regression model was used to accurately determine the predictors, which showed that behavioral confidence and changes in the physical environment are significant predictors for the initiation of breast self-awareness (ADJ. *R*^2^ = 0.192). practice for change and changes in the social environment significantly affected the maintenance of breast self-awareness behavior (ADJ. *R*^2^ = 0.334).

In the initiation model, behavioral confidence (β = 0.251, *p* = 0.010) and changes in the physical environment (β = 0.256, *p* = 0.012) showed significant positive associations with intention to initiate breast self-awareness, indicating that higher levels of confidence and better access to physical resources were associated with stronger initiation intentions. Participatory dialogue did not reach statistical significance and was therefore excluded from the final stepwise model (Table 3). Regarding maintenance of breast self-awareness, practice for change emerged as the strongest predictor (β = 0.449, *p* < 0.001), followed by changes in the social environment (β = 0.220, *p* = 0.008). Emotional transformation was not retained in the final model, suggesting a limited role in sustaining breast self-awareness behavior in this population (Table 4). The correlation between initiation and maintenance constructs of breast self-awareness is shown in Table 5. Significant positive correlations were observed among key constructs, particularly behavioral confidence and physical environment in the initiation model, and emotional transformation, practice for change, and social environment in the maintenance model (*p* < 0.001).

**Table 3:** Multiple linear regression analysis predicting initiation of breast self-awareness behavior.

Predictor variables	B	SE	Beta	*t*	*p*-Value
Constant	1.152	0.267	—	5.467	<0.001
Behavioral confidence	0.053	0.021	0.251	2.563	0.010
Physical environment changes	0.095	0.036	0.256	2.610	0.012
*R* = 0.547	*R*^2^ = 0.205		Adjusted *R*^2^ = 0.192		

B, unstandardized coefficient; SE, standard error; beta, standardized coefficient. The model was fitted using stepwise multiple linear regression. Significance was considered at *p* < 0.05.

**Table 4:** Multiple linear regression analysis predicting maintenance of breast self-awareness behavior.

Predictor variables	B	SE	Beta	*t*	*p*-Value
Constant	1.027	0.272	—	3.772	<0.001
Practice for change	0.161	0.029	0.449	5.491	<0.001
Social environment changes	0.077	0.029	0.220	2.693	0.008
*R* = 0.587	*R*^2^ = 0.345		Adjusted *R*^2^ = 0.334		

B, unstandardized coefficient; SE, standard error; beta, standardized coefficient. The model was fitted using stepwise multiple linear regression. Significance was considered at *p* < 0.05.

**Table 5:** Correlation between the constructs of initiation and maintenance of breast self-awareness.

Variables	1	2	3	4	5	6	7	8
Initiation constructs								
1. Intention to initial breast self-awareness	1	*r* = 0.003 *p*= 0.255	*r* = 0.403^**^p < 0.000	*r* = 0.404^**^p < 0.000	*r* = 0.565^**^p < 0.000	*r* = 0.589^**^p < 0.000	*r* = 0.523^**^p < 0.000	*r* = 0.419^**^p < 0.000
2. Participatory dialogue	—	1	*r* = 0.247^**^p = 0.005	*r* = 0.347^**^p = 0.000	*r* = 0.225^**^p = 0.010	*r* = 0.366^**^p = 0.000	*r* = 0.219^**^p = 0.012	*r* = 0.500^**^p < 0.001
3. Behavioral confidence	—	—	1	*r* = 0.591^**^p < 0.000	*r* = 0.276^**^p < 0.001	*r* = 0.673^**^p < 0.000	*r* = 0.547^**^p < 0.000	*r* = 0.373^**^p < 0.001
15.5-7.4,-13.5175.3mm 4. Change in the physical environment	—	—	—	1	*r* = 0.431^**^p < 0.000	*r* = 0.720^**^p < 0.000	*r* = 0.639^**^p < 0.000	*r* = 0.581^**^p < 0.000
Maintenance constructs								
5. Intention to maintain breast self-awareness	—	—	—	—	1	*r* = 0.532^**^p < 0.000	*r* = 0.554^**^p < 0.000	*r* = 0.435^**^p < 0.000
6. Emotional transformation	—	—	—	—	—	1	*r* = 0.748^**^p < 0.000	*r* = 0.525^**^p < 0.000
7. Practice for change	—	—	—	—	—	—	1	*r* = 0.478^**^p < 0.001
8. Changes in the social environment	—	—	—	—	—	—	—	1

^**^At the initiation of behavior change, the constructs of behavioral confidence and change in the physical environment are correlated, while at the maintenance of behavior change, the constructs of emotional transformation, practice for change, and social environment changes are correlated.

## Discussion

To the best of our knowledge, this study is among the first to apply the Multi-Theory Model (MTM) to examine factors associated with initiation and maintenance of breast self-awareness among female health workers in Kabul, Afghanistan. Given the increasing burden of breast cancer in Afghanistan (1) and the absence of organized national screening programs (38), breast self-awareness represents a feasible, low-cost preventive strategy (39). In this sociocultural context—where late-stage diagnosis remains common (40) and access to mammography is limited (40), understanding behavioral determinants among female health workers is especially important, as they function both as healthcare providers and as trusted sources of information for women in their communities (41).

The findings showed that behavioral confidence associated with initiation of breast self-awareness. This is consistent with previous MTM-based studies in preventive health behaviors, such as mammography screening (42), small portion size consumption (23), and smoking cessation (43). These studies collectively suggest that self-belief may play an important role of self-belief in initiating health behavior change within the MTM framework. However, behavioral confidence may be particularly important in the Afghan context. Cultural sensitivity surrounding breast-related discussions, modesty norms, and limited structured education on breast health may partially contribute to reduced confidence among women in identifying normal vs. abnormal changes (44). Evidence suggests that even among health workers, knowledge and practice of breast self-awareness may remain suboptimal, indicating potential gaps in formal training (45). Therefore, strengthening behavioral confidence through structured educational workshops, skill-based demonstrations, and culturally appropriate training modules may be critical for improving initiation rates in Afghanistan (46).

Changes in the physical environment also significantly associated with initiation of breast self-awareness. Similar findings have been reported in MTM-based research examining sugar-sweetened beverage reduction (31). Physical activity (25), and mammography screening (32), where environmental facilitation improved the likelihood of initiating behavior change. However, in Afghanistan, environmental barriers are more pronounced. Limited availability of female healthcare providers, lack of private examination spaces, inconsistent referral systems, and financial constraints may act as barriers to preventive practices (47). Even female health workers may experience structural barriers that could influence their personal preventive behaviors (48). Thus, interventions in Afghanistan must address not only cognitive and motivational factors but also healthcare infrastructure and accessibility (49).

Participatory dialogue did not significantly predict initiation in this study. While some MTM-based studies—such as those on waterpipe smoking reduction (50) and physical activity (51) have identified participatory dialogue as a significant predictor, in a study by Lauen Brown on vegetable and fruit consumption, non-significant findings were reported for this construct (52). In the context of Afghanistan, Cultural norms and taboos surrounding breast health may limit open discussion between health educators and female health workers and may reduce the effectiveness of participatory dialogue. This suggests that communication channels, skills, and culturally sensitive approaches may play an important role to fully engage participants in discussions about breast self-awareness. Limited open discussions about breast health, especially in mixed or conservative settings, may reduce opportunities for meaningful dialogue about advantages and disadvantages. Social taboos and discomfort in discussing breast-related topics may weaken this construct (40). Therefore, future interventions should incorporate culturally sensitive, women-only discussion platforms to enhance participatory dialogue (40).

Regarding maintenance of behavior, practice for change emerged as the strongest associated factor. This finding aligns with prior MTM studies examining sustained behaviors such as physical activity (28), fruit and vegetable consumption (52), and mammography screening (32). The consistent role of self-regulation strategies within the MTM framework underscores the importance of habit formation and behavioral planning in long-term maintenance (53). In Afghanistan, this finding is particularly relevant. Women in this context may often manage multiple responsibilities, including professional duties and family caregiving (54–56). Preventive health behaviors may be relatively deprioritized when daily survival needs dominate attention (57). Establishing reminder systems, integrating breast self-awareness into weekly routines, and embedding it within professional healthcare protocols may improve sustainability (58). Institutional reinforcement within healthcare centers could further strengthen adherence among female staff (59, 60).

Changes in the social environment also significantly associated with maintenance of breast self-awareness. Previous MTM studies on binge drinking (24), smoking cessation (43), adequate sleep (61), and mammography screening (42) similarly identified social support as a key determinant of sustained behavior. Afghan society is highly family-oriented and community-driven; health behaviors are strongly influenced by social norms and family approval (62). Support from spouses, colleagues, supervisors, and extended family members may play an important role in reinforcing preventive practices (63). Encouraging supportive workplace cultures within healthcare facilities and engaging family members in awareness programs may therefore enhance long-term maintenance (64).

Interestingly, emotional transformation was not retained as a significant predictor of maintenance. While several MTM-based studies in other contexts have demonstrated its predictive value [e.g., sleep (61), sugar-sweetened beverages (31), HPV vaccination (65), physical activity (66)], its lack of significance here may reflect competing psychosocial stressors faced by Afghan women (56). Ongoing economic challenges, security concerns, and healthcare system instability may limit the emotional prioritization of preventive behaviors (56). Breast self-awareness may not yet be fully internalized as an emotionally salient health goal (48). Future interventions may benefit from framing breast health within broader narratives of women's empowerment, maternal responsibility, and family wellbeing to increase emotional engagement (67).

### Strengths and weaknesses of the study

This study has several strengths, notably the use of the Multi-Theory Model (MTM) of health behavior change, a relatively novel model with unique constructs, to examine breast self-awareness among female health workers. Despite these strengths, several limitations should be acknowledged. First, the cross-sectional design limits causal inference. Second, the study was conducted in a limited geographical and social setting (western Kabul) using convenience sampling, which may introduce selection bias and limit the generalizability of the findings beyond the study population. Future studies using probability sampling methods are recommended. Third, although the sample size (*n* = 130) exceeded the minimum required sample size (*n* = 108) calculated for examining individual regression coefficients, it may still limit statistical power and model stability, particularly for multivariable regression analyses. However, stepwise regression was applied to identify the most relevant predictors and reduce the risk of overfitting. Future studies with larger sample sizes are recommended to enhance model stability and allow simultaneous inclusion of all MTM constructs. Forth, the use of self-reported data may introduce recall and social desirability bias. To minimize these effects, data were collected anonymously in a private setting without the presence of supervisors or administrative staff. Future studies incorporating objective or observational measures are recommended. Fifth, the questionnaire was only assessed for face validity and internal consistency and expert review; further psychometric validation, including construct validity and test-retest reliability, was not conducted, which may introduce measurement bias and affect replicability. Future studies are recommended to conduct full psychometric evaluations to strengthen the validity and reliability of the measurement tool. Sixth, adjusted *R*^2^ values indicate that 67%−81% of the variance in breast self-awareness behavior remains unexplained, suggesting that other factors outside MTM may influence behavior. Finally, cultural and contextual factors in Afghanistan, including potential taboos around breast health, may have affected responses and the non-significance of participatory dialogue.

### Future directions

Given the limitations of the current study design, future research should prioritize the use of randomized controlled trials (RCTs) to evaluate the effectiveness of interventions. These studies could provide stronger evidence of the predictive power of the Multi-Theory Model (MTM) constructs in different settings. In addition, the inclusion of health professionals from diverse regions could contribute to a more comprehensive understanding of breast self-awareness and behavior change in larger populations.

### Implications for future interventions

The findings of this study have practical implications for health education and health promotion. The identification of behavioral confidence, physical environment, practice for change, and social environment as key predictors suggests that theory-based educational interventions focusing on skill development, supportive environments, and sustained engagement may improve breast self-awareness among female health workers. These findings may also inform policy-level breast cancer prevention strategies, particularly in resource-limited settings. Based on the results of this study, future breast self-awareness interventions should focus on strengthening key constructs of the Multi-Theory Model (MTM) of health behavior change, particularly constructs such as behavioral confidence and changes in the physical and social environment. These constructs have played an important role in predicting the initiation and maintenance of breast self-awareness behaviors. In addition, given the contextual differences in different communities, interventions should be specifically tailored to the cultural, social, and economic characteristics of each region. In some areas, there may be a need to improve infrastructure and educational resources to facilitate the breast self-awareness process. Therefore, future research should focus not only on evaluating the effectiveness of these constructs but also on better understanding how they are influenced by different settings and designing personalized interventions.

### Contextual differences

Because this study was conducted in the western region of Kabul and focused on female health workers, its results should be interpreted with caution in the context of cultural, social, and economic differences in other countries and regions. In particular, the results of this study may differ from those of similar studies conducted in countries such as the United States or Nepal due to various contextual differences. For example, cultural norms, social support systems, and access to health care resources may have a significant impact on the effectiveness of breast self-awareness and behavior change interventions. Therefore, future research should specifically address these contextual differences and aim to gain a more comprehensive understanding of how the constructs of the Multi-Theory Model (MTM) operate in different settings and how they influence diverse populations.

### Overall conclusion

Application of the Multi-Theory Model (MTM) of health behavior change among healthcare workers in the western region of Kabul indicated that two constructs were significantly associated with initiation of breast self-awareness, and two other constructs were significantly associated with maintenance of behavior change. Behavioral confidence and changes in the physical environment were associated with initiation, while practice for change and changes in the social environment were associated with maintenance. These findings provide preliminary evidence supporting potential associations between MTM constructs and breast self-awareness behaviors in this population. However, due to the cross-sectional design and other methodological limitations, causal interpretations cannot be made. Further research is needed to confirm these associations and explore their applicability in different contexts.

## Data Availability

The raw data supporting the conclusions of this article will be made available by the authors, without undue reservation.
